# Novel Bioinspired Approach Based on Chaotic Dynamics for Robot Patrolling Missions with Adversaries

**DOI:** 10.3390/e20050378

**Published:** 2018-05-18

**Authors:** Daniel-Ioan Curiac, Ovidiu Banias, Constantin Volosencu, Christian-Daniel Curiac

**Affiliations:** 1 Automation and Applied Informatics Department, Politehnica University of Timisoara, 300223 Timisoara, Romania; 2 Electrical Engineering and Information Technology Department, Technische Universität München, 80333 Munich, Germany

**Keywords:** positional entropy, protean behavior, mobile robot, adversarial patrolling, chaotic dynamics, Arnold’s cat map

## Abstract

Living organisms have developed and optimized ingenious defense strategies based on positional entropy. One of the most significant examples in this respect is known as protean behavior, where a prey animal under threat performs unpredictable zig-zag movements in order to confuse, delay or escape the predator. This kind of defensive behavior can inspire efficient strategies for patrolling robots evolving in the presence of adversaries. The main goal of our proposed bioinspired method is to implement the protean behavior by altering the reference path of the robot with sudden and erratic direction changes without endangering the robot’s overall mission. By this, a foe intending to target and destroy the mobile robot from a distance has less time for acquiring and retaining the proper sight alignment. The method uses the chaotic dynamics of the 2D Arnold’s cat map as a primary source of positional entropy and transfers this feature to every reference path segment using the kinematic relative motion concept. The effectiveness of this novel biologically inspired method is validated through extensive and realistic simulation case studies.

## 1. Introduction

Starting from the classical Shannon’s definition of information entropy as a measure of uncertainty in a discrete distribution [1,2], Pal and Pal [3] proposed a new concept that characterizes the localization uncertainty of a particular object in a given scene: *positional entropy*. This type of entropy basically quantifies the navigational unpredictability of an object [4,5] and has higher values in the case of random and unexpected movements [4,5,6] as the ones that characterize the protean behavior of diverse animal species under threat.

Throughout their evolution over millions of years, living organisms have developed ingenious defense mechanisms to protect themselves against enemies, including camouflage, mimicry, fleeing, hiding, freezing, etc. Of a special interest for robotic path planning in adversarial environments is a particular defense strategy, employing positional entropy, named protean behavior [7,8,9]. Here, a prey animal under threat pursues an unpredictable zig-zag path, with sudden direction and speed changes meant to confuse, delay and escape the predator. As a consequence, the protean behavior is proved to be effective not only as a response to an ongoing attack, but also as a prevention measure for various animal species including voles and spiny mice [10], cockroaches [11], hares or minnows [12].

When accomplishing patrol missions, mobile robots may face two types of attacks: (a) adversary (e.g., a sniper) may target and destroy the robots from a distance; or, in rare situations; and (b) adversary may chase and capture the robots. From our perspective, imparting protean behavior to patrol robots can efficiently countermeasure the first type of attacks by offering the enemies less time to acquire and retain the proper sight alignment, while in the case of mobile robots chased by enemies, protean behavior can be envisioned only as a complementary method. In both cases, such strategies may be feasibly implemented within an intelligent path planning mechanism. 

Traditionally, the path planning problem in adversarial conditions has been tackled following two different lines of attack: the game theoretic approach; and the path chaotization to ensure higher levels of path unpredictability.

In game theory, the robot motion in the presence of adversaries is basically interpreted as a pursuit-evasion game [13,14] where based on three models (i.e., mathematical models of prey, predator and environment) diverse path planning strategies are implemented. As a result, a series of relevant studies have been reported for different settings involving single-robot [15], multi-robot [16,17], or swarm robotic systems [18] with full or limited information about adversaries [19,20]. An interesting approach in this context is presented in [21] where the protean fleeing is envisioned as a possible solution. However, such game theoretic approaches, even very theoretically challenging and extremely useful for gaining a profound understanding of the real-life scenarios, are still far from being implemented on autonomous robots due to their numerous simplifying assumptions, imprecision and uncertainties associated with the employed mathematical models and computational complexity [22,23].

The second stratagem to cope with opponents is based on the reasonable assumption that an unpredictable path can help securing the robot patrol mission. Derived from the inherent long-term unpredictability provided by chaotic systems due to their sensitivity to the initial conditions characteristics [24,25,26], these types of approaches cover a large variety of patrolling missions including point of interest surveillance [27,28], perimeter surveillance [29] and area surveillance [30,31,32,33,34]. When speaking about short-term unpredictability that characterizes the protean behavior, the direct use of chaotic dynamics is inefficient but discrete chaotic systems may offer the needed source of path randomness. In this context, in the only reported method to date that tackles the protean behavior [35], we employed the inherent robot’s obstacle avoidance mechanism to circumvent chaotically generated Fictive-Temporary Obstacles (FTOs) in the attempt to provide the needed irregular zigzagging path. While the mentioned method utilizes a combined software-hardware mechanism that changes both the path planning algorithm and the robot’s obstacle sensing module, in this paper, we tackle the problem from a different angle, the zig-zag movements being generated using only software means (only the path planning algorithm is altered).

In this paper, we provide a biologically inspired method to address the scenarios where the adversaries target the patrol robot from a distance. We start with analyzing the 2D Arnold’s cat map (ACM), a simple discrete chaotic system that provides random-like sequences of points. Using the kinematic relative motion concept, the randomness provided by ACM is utilized to change every segment of the reference path into a misleading zig-zag trajectory, thus imparting the required positional entropy by emulating the protean behavior of prey animals under threat. For this, a specially tailored Additional Waypoints Generation (AWG) algorithm is designed. The effectiveness of the AWG-based method is validated in realistic simulation case studies, proving certain advantages over FTO-based method in obstacle-free environments or in environments with small or medium size obstacles. If large obstacles are encountered, the paper suggests a mixture of the two methods.

The rest of the paper is organized as follows. Section 2 presents the problem formulation, giving some preparatory analysis about its future solving. While Section 3 is devoted to the design of additional waypoints generation algorithm, in Section 4, the bioinspired patrolling method is described, offering detailed implementation insights. Section 5 provides two extensive simulation case studies that validate the effectiveness of the proposed method and its advantages over the FTO-based method. In the final section, conclusions and some final remarks are given.

## 2. Problem Formulation

As proved by various animal species, the protean behavior is an efficient tactic when coping with adversaries [7,8,9]. Imparting such a behavior to mobile robots accomplishing patrol missions presume sudden direction changes without affecting their long-term assignment. In these conditions, the robot’s path planning mechanism will alter its normal trajectory into a misleading path for enemies. By this, the opponents trying to target and destroy the robot from a distance will have less time for acquiring and retaining the proper sight alignment. Our strategy is based on inserting supplementary waypoints to obtain small zig-zag movements that emulate the behavior of individual prey animal under threat.

***Problem*** ***statement***: *Let us consider an autonomous mobile robot that accomplishes a given patrolling task in a two-dimensional workspace $W\subset {\mathbb{R}}^{2}$ containing a set of n fixed obstacles O_i_, i = 1, …, n. The robot is outfitted with onboard sensing and computation capabilities. The assignment is to adjust the robot’s path using only onboard capabilities in order to cope with adversaries, without jeopardizing its basic mission.*

In [35], a similar problem is solved by employing a combined hardware-software method that simulates sequences of fictive and temporary obstacles. In this paper, we target a pure software solution for this problem that will change only the path planning algorithm. Our idea is to utilize the chaotic dynamics of 2D Arnold’s Cat Map to generate additional waypoints without significantly modifying the overall shape of the trajectory. By this, we ensure the required short-term unpredictability of the robot path in adversarial conditions, similar to the protean behavior of some species of prey animals when being chased by enemies. 

The proposed approach may be straightforwardly implemented on mobile robots evolving in unknown environments and endorsed with suitable obstacle avoidance mechanisms (e.g., bug-type algorithms like Bug0, OneBug, Distbug, LeaveBug and TangentBug [36]). If the autonomous robots are equipped with enough onboard computational power, they may handle the processing of temporary maps, and, by this, the proposed technique may be implemented even in combination with global path planning algorithms like Breadth-first search, Depth-first search, A*, Dijkstra’s algorithm, or Potential Field [37].

## 3. Additional Waypoints Generation Algorithm

### 3.1. Preliminaries

Let *P_i_*(*x_i_*, *y_i_*) and *P_i_*_+1_(*x_i+_*_1_, *y_i_*_+1_) denote two successive waypoints of the robot trajectory. Our task is to replace the optimal line segment with a zig-zag path to impart short-term unpredictability to robot motion. This protean behavior is produced by a sequence of *M_i_* newly generated supplementary waypoints that are included in the robot path from *P_i_* to *P_i_*_+1_. We will denote these supplementary waypoints with *Q_im_*, with *m =* 1, 2, …, *M_i_*.

In general, two different ways can be pursued to obtain the sequence of random-looking waypoints: to use a stochastic approach based on a random number generator to produce a kind of random-walk; or to use a pure deterministic approach employing a chaotic system. When involved in a patrol mission, the location of the autonomous robot must be unpredictable for opponent entities but known to ally entities (e.g., mission coordinators or other robots from the same team). In the case of a deterministic approach ally entities, having complete information about the robot’s path planning mechanism, may recalculate the robot trajectory and later use this knowledge for decision-making purposes. Based on this requirement, our proposed AWG algorithm will provide the sequence of waypoints in a deterministic fashion using Arnold’s cat map, a 2D chaotic system that offers simplicity in terms of required computations and a random-like distribution of waypoints within the unit square [38].

### 3.2. Arnold’s Cat Map

The additional waypoints will be generated such as the robot trajectory inside a specified zone to appear random and unpredictable for adversaries or external observers. For this, we utilized the inherent unpredictability provided by any chaotic system due to its sensitivity to the initial conditions property [24,25,26]. An effective solution in this respect is to employ the discrete chaotic dynamics provided by the hyperbolic toral automorphism, known as Arnold’s cat map [39,40]. This discrete 2D map is one of the most investigated examples of dynamical systems mainly due to its broad range of applications in image encryption and watermarking [41]. It is described by the following equations:

(1) $$\{\begin{array}{l} {\hat{x}}_{j}=\left({\hat{x}}_{j-1}+{\hat{y}}_{j-1}\right)mod1\\ {\hat{y}}_{j}=\left({\hat{x}}_{j-1}+2\cdot {\hat{y}}_{j-1}\right)mod1 \end{array}$$

Here, the *mod*1 operator (modulo-1) limits the ${\hat{x}}_{j}$ and ${\hat{y}}_{j}$ state variables to the interval [0, 1). On ℝ^2^∖ℤ^2^, the transformation (1) describes an obvious chaotic behavior [42] shown by its maximal Lyapunov exponent $\lambda_{1}=ln\left(0.5\cdot \left(3+\sqrt{5}\right)\right)=0.9624>0$, while the other Lyapunov exponent is $\lambda_{2}=ln\left(0.5\cdot \left(3-\sqrt{5}\right)\right)=-0.9624<0$. Another important characteristic of this map is that the determinant of its linear part is equal to 1, demonstrating that the transformation is area-preserving and, of course, invertible [42].

### 3.3. Emulating Zig-Zag Movements

**Figure 1:**
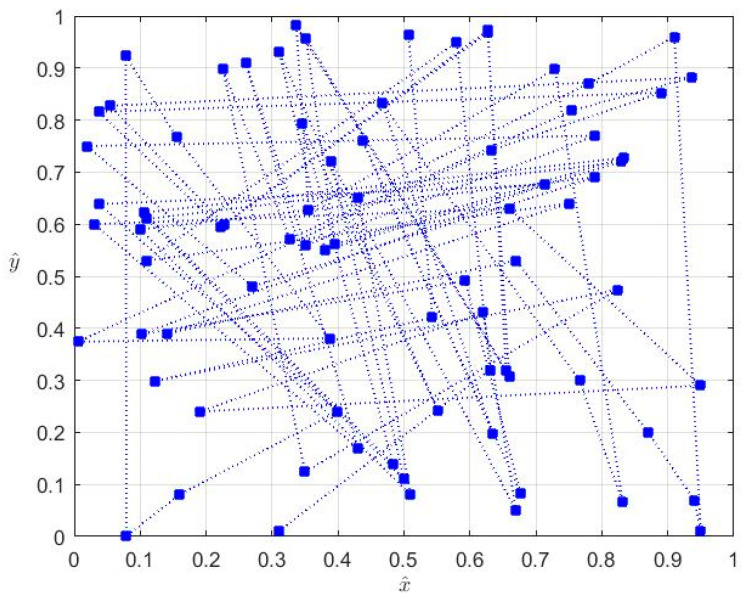
Arnold’s cat map.

The starting point of our approach is the graphical representation of Arnold’s cat map (Figure 1), a discrete chaotic system that generates a sequence of random-looking points inside the unit square.

**Figure 2:**
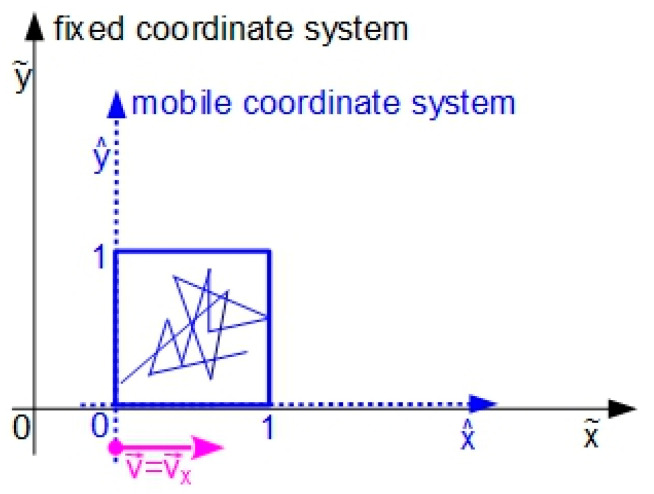
Obtaining the compound motion.

In order to emulate the protean behavior, we will employ a simple kinematic concept—relative motion [43]. This concept is used to translate the representation of a motion developed inside a mobile coordinate system into coordinates of a fixed coordinate system. Our stratagem can be described by the following situation: we consider two Carthesian coordinate systems: (a) one fixed; and (b) one mobile in which the ACM evolves. The two coordinate systems have collinear horizontal axes, and the mobile one, where the ACM evolves, is dragged with a constant speed $\vec{v}$ along the horizontal axis of the fixed coordinate system (Figure 2).

Such a compound motion will be described by the following set of equations:

(2) $$\{\begin{array}{l} {\hat{x}}_{j}=\left({\hat{x}}_{j-1}+{\hat{y}}_{j-1}\right)mod1\\ \begin{array}{l} {\hat{y}}_{j}=\left({\hat{x}}_{j-1}+2\cdot {\hat{y}}_{j-1}\right)mod1\\ {\tilde{x}}_{j}={\hat{x}}_{j}+v\cdot j \end{array}\\ {\tilde{y}}_{j}={\hat{y}}_{j} \end{array}$$

where the first two equations represent the trajectory described by ACM in the mobile system $\left(\hat{x},\hat{y}\right),$ while the other two equations describe the coordinate translation from mobile to the fixed coordinate system $\left(\tilde{x},\tilde{y}\right).$

**Figure 3:**
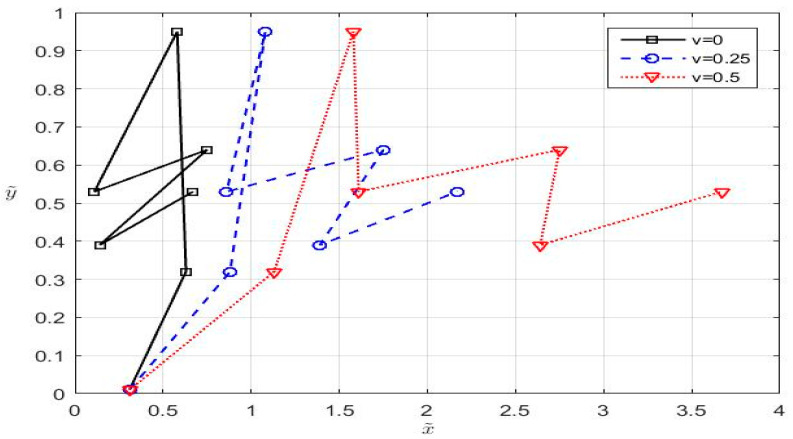
The compound motion inside the fixed coordinate systems for different values of *v*.

By this, a compound motion that emulates the zig-zag shape, which characterizes a protean behavior is obtained. To have a more realistic perspective about this transformation of ACM, we plotted the obtained trajectory for *v* = 0, *v* = 0.25 and *v* = 0.5 in Figure 3.

### 3.4. Adapting the Zig-Zag to a General Segment of Robot Trajectory

**Figure 4:**
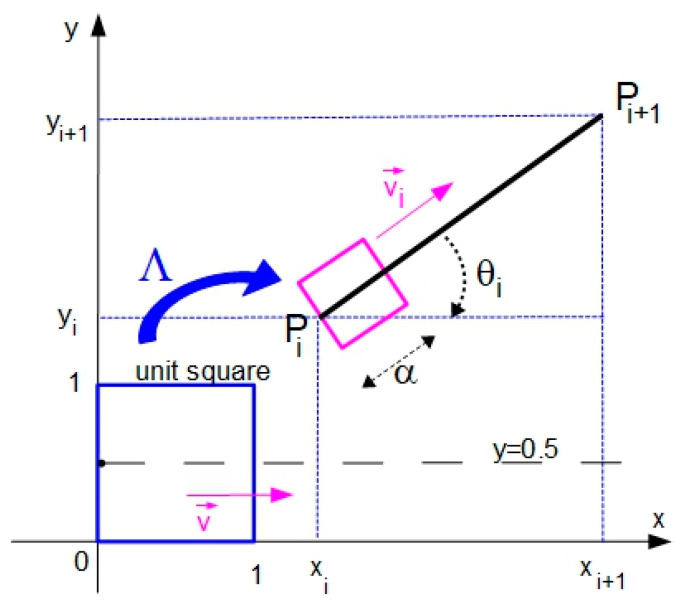
Adapting the motion to a general segment *P_i_P_i_*_+1_.

By visually analyzing Figure 3 and knowing the feature of ACM to provide random-like points inside the unit square [38], we can state that the strategy presented in the last subsection can provide random waypoints around the $\tilde{y}=0.5$ line, starting with (0, 0.5). If we want to provide additional waypoints around a general *P_i_P_i_*_+1_ segment, the system (2) must be adapted using a compound affine transform Λ, while the vector ${\vec{v}}_{i}$ will have the same direction as the vector $\vec{P_{i}P_{i+1}}$ with components along both axes, as depicted in Figure 4. These required changes will affect only the last two equations of (2).

The affine transform Λ used to modify the coordinates provided by (1) is composed of a translation, a scaling and a rotation and has the following transformation matrix (in homogeneous coordinates):

(3) $$T_{\Lambda }=T*S*R=\left[\begin{array}{ccc} 1 & 0 & x_{i}+0.5\cdot \alpha \cdot \sin\left(\vartheta_{i}\right)\\ 0 & 1 & y_{i}-0.5\cdot \alpha \cdot \cos\left(\vartheta_{i}\right)\\ 0 & 0 & 1 \end{array}\right]\left[\begin{array}{ccc} \alpha & 0 & 0\\ 0 & \alpha & 0\\ 0 & 0 & 1 \end{array}\right]\left[\begin{array}{ccc} \cos\left(-\vartheta_{i}\right) & \sin\left(-\vartheta_{i}\right) & 0\\ -\sin\left(-\vartheta_{i}\right) & \cos\left(-\vartheta_{i}\right) & 0\\ 0 & 0 & 1 \end{array}\right]$$

where $\vartheta_{i}=\mathrm{atan}\left(\frac{y_{i+1}-y_{i}}{\;x_{i+1}-x_{i}}\right)$ and α is a constant that characterizes the area where the points will be spread.

Using (3), the transformation Λ of a point generated by ACM inside unit square and having the coordinates $\left({\hat{x}}_{j},{\hat{y}}_{j}\right)$ into a point inside a general square (Figure 4) can be written as follows:

(4) $$\left[\begin{array}{c} x_{j}\\ y_{j}\\ 1 \end{array}\right]=\left[\begin{array}{ccc} \alpha \cdot \cos\left(-\vartheta_{i}\right) & \alpha \cdot \sin\left(-\vartheta_{i}\right) & x_{i}+0.5\cdot \alpha \cdot \sin\left(\vartheta_{i}\right)\\ -\alpha \cdot \sin\left(-\vartheta_{i}\right) & \alpha \cdot \cos\left(-\vartheta_{i}\right) & y_{i}-0.5\cdot \alpha \cdot \cos\left(\vartheta_{i}\right)\\ 0 & 0 & 1 \end{array}\right]\left[\begin{array}{c} {\hat{x}}_{j}\\ {\hat{y}}_{j}\\ 1 \end{array}\right]$$

or

(5) $$\{\begin{array}{l} x_{j}=\alpha \cdot \cos\left(-\vartheta_{i}\right)\cdot {\hat{x}}_{j}+\alpha \cdot \sin\left(-\vartheta_{i}\right)\cdot {\hat{y}}_{j}+x_{i}+0.5\cdot \alpha \cdot \sin\left(\vartheta_{i}\right)\\ y_{j}=-\alpha \cdot \sin\left(-\vartheta_{i}\right)\cdot {\hat{x}}_{j}+\alpha \cdot \cos\left(-\vartheta_{i}\right)\cdot {\hat{y}}_{j}+y_{i}-0.5\cdot \alpha \cdot \cos\left(\vartheta_{i}\right) \end{array}$$

In order to obtain the adapted versions of the last two equations from (2), we have to integrate in (5) the effect produced by dragging the mobile system with the constant speed vector ${\vec{v}}_{i}$ that has the same direction and orientation as the vector $\vec{P_{i}P_{i+1}}$. By this, we obtain:

(6) $$\{\begin{array}{l} x_{j}=\alpha \cdot \cos\left(-\vartheta_{i}\right)\cdot {\hat{x}}_{j}+\alpha \cdot \sin\left(-\vartheta_{i}\right)\cdot {\hat{y}}_{j}+x_{i}+0.5\cdot \alpha \cdot \sin\left(\vartheta_{i}\right)+v_{i,x}\cdot j\\ y_{j}=-\alpha \cdot \sin\left(-\vartheta_{i}\right)\cdot {\hat{x}}_{j}+\alpha \cdot \cos\left(-\vartheta_{i}\right)\cdot {\hat{y}}_{j}+y_{i}-0.5\cdot \alpha \cdot \cos\left(\vartheta_{i}\right)+v_{i,y}\cdot j \end{array}$$

where $v_{i,x}$ and $v_{i,y}$ are the projections of the speed vector ${\vec{v}}_{i}$ (associated with the vector $\vec{P_{i}P_{i+1}}$) on the axes, which can be either positive or negative. By replacing the last two equations from (2) with equations provided by (6), we will obtain the final form of the system used to generate *M_i_* additional waypoints:

(7) $$\left\{ \begin{array}{l} {\hat{x}}_{j}=\left({\hat{x}}_{j-1}+{\hat{y}}_{j-1}\right)mod1\\ {\hat{y}}_{j}=({\hat{x}}_{j-1}+2\cdot {\hat{y}}_{j-1})mod1\\ x_{j}=\alpha \cdot \cos\left(-\vartheta \right)\cdot {\hat{x}}_{j}+\alpha \cdot \sin\left(-\vartheta \right)\cdot {\hat{y}}_{j}+x_{i}+0.5\cdot \alpha \cdot \sin\left(\vartheta \right)+v_{i,x}\cdot j\\ x_{j}=-\alpha \cdot \sin\left(-\vartheta \right)\cdot {\hat{x}}_{j}+\alpha \cdot \cos\left(-\vartheta \right)\cdot {\hat{y}}_{j}+y_{i}-0.5\cdot \alpha \cdot \cos\left(\vartheta \right)+v_{i,y}\cdot j \end{array}with\;j=1\ldots M_{i}\right.$$

**Figure 5:**
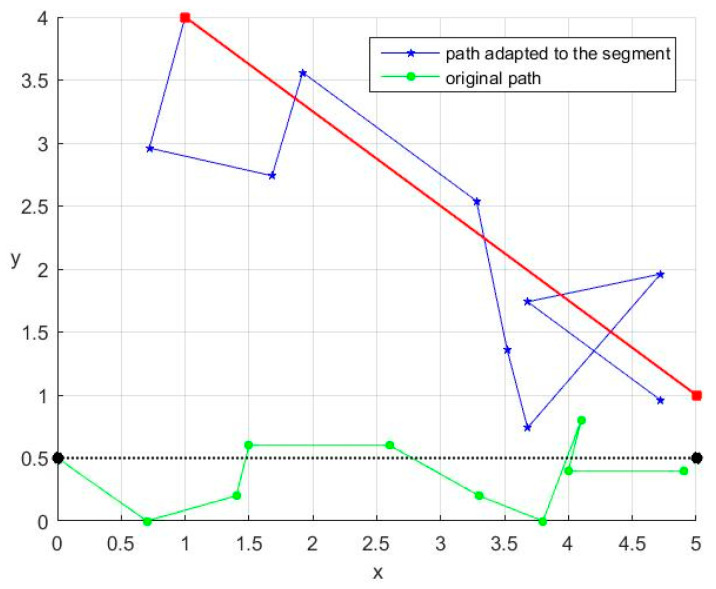
Adapting the path to *P_i_P_i+_*_1_ segment.

Figure 5 presents the path adaptation to the *P_i_P_i+_*_1_ segment, where *P_i_*(1, 4) and *P_i_*_+1_(5, 1), when α=2 and the magnitude of speed vector *v* = 0.5:

For a given segment *P_i_P_i_*_+1_ of the reference path, the initialization of system (7) includes the selection of the following parameters: initialization values for ACM (${\hat{x}}_{0},{\hat{y}}_{0})$; the number *M_i_* of additional waypoints generated for the segment *P_i_P_i_*_+1_; the constant α that characterizes the spreading of the additional waypoints around the segment *P_i_P_i_*_+1_ (each supplementary waypoint will be generated within an α/2 distance from the unaltered path as described by Figure 5); and the magnitude of speed vector ${\vec{v}}_{i}$. Details about the selection of these values will be given in Section 4.

### 3.5. Pseudocode of AWG Algorithm

Based on (7), we can provide the additional waypoints that will alter the segment between two successive waypoints *P_i_* and *P_i_*_+1_ of initial trajectory using the following pseudocode for the AWG algorithm:

1. [*Q*_*i*,1_, …*Q_i,M_*,${\hat{x}}_{M_{i}}$, ${\hat{y}}_{M_{i}}$] = AWG (*P_i_*, *P*_*i*+1_, *M_i_*,${\hat{x}}_{0}$, ${\hat{y}}_{0},$*v*,α,) 2. { 3.  $\vartheta_{i}=\mathrm{atan}\left(\frac{y_{i+1}-y_{i}}{\;x_{i+1}-x_{i}}\right)$; 4.  $v_{i,x}=sgn\left(x_{i+1}-x_{i}\right)\cdot \left|v\cdot \cos\left(\vartheta_{i}\right)\right|$; 5.  $v_{i,y}=sgn\left(y_{i+1}-y_{i}\right)\cdot \left|v\cdot \sin\left(\vartheta_{i}\right)\right|$; 6.  **for***j* = 1 to *M_i_* 7.   iterate system (7); 8.  **return***Q*_*i*,1_, …*Q_i,M_*, ${\hat{x}}_{M_{i}}$, ${\hat{y}}_{M_{i}}$; 9. }

The function AWG receives seven parameters (the coordinates of the two successive waypoints *P_i_* and *P_i+_*_1_; the number *M_i_* of additional waypoints that must be generated for *P_i_P_i_*_+1_ segment; the initialization values for ACM (${\hat{x}}_{0}$, ${\hat{y}}_{0}$); the magnitude of speed *v*; and the value α that characterizes the spreading of the additional waypoints) and provides the sequence of additional waypoints for *P_i_P_i_*_+1_ segment (*Q_i_*_,*j*_ with *j* = 1, *…*, *M_i_*) and the last coordinates inside the unit square obtained by ACM (to be used as initial values for ACM generator for the following segment). The pseudocode starts by calculating the angle $\vartheta_{i}$ and the components of vector ${\vec{v}}_{i}$ along axes (*v_i_*_,*x*,_ *v_i_*_,*y*_). After that, system (7) is iterated to provide the coordinates of the additional waypoints *Q_i_*_,1_, …, *Q_i_*_,*Mi*_ and the values for the coordinates of the last point offered by ACM (${\hat{x}}_{M}$, ${\hat{y}}_{M}$)—these values will be used to initialize the ACM (first two equations of (7)) when computing the additional waypoints for the following segment (*P_i_*_+1_*P_i+_*_2_).

## 4. Patrolling Method Implementation

Let us consider a general case where the mobile robot is tasked with a patrol mission described by a sequence of waypoints *P_i_*, with *i =* 1, …, *N*, and the corresponding maximum time *t_max_* to accomplish this mission. Based on the AWG function presented above, we can split the implementation of the bioinspired robot motion mechanism in three stages: initialization, offline computations and mission accomplishment.

**Phase 1: Initialization**

This stage includes the selection of the following parameters:

- set of fixed waypoints along the unaltered route (*P_i_*, *i* = 1, …, *N*) and the maximum time to accomplish the mission (*t_max_*); These parameters are the basic descriptors of the robot’s patrolling mission and their selection is closely linked with mission’s objectives and constraints.
- initial values for ACM $\left({\hat{x}}_{0},{\hat{y}}_{0}\right)$; As mentioned in Section 3.2, we must choose $\left({\hat{x}}_{0},{\hat{y}}_{0}\right)\in {\mathbb{R}}^{2}\setminus {\mathbb{Z}}^{2}$ to obtain a chaotic behavior, an appropriate selection being $\left({\hat{x}}_{0},{\hat{y}}_{0}\right)\in \left(0,1\right)x\left(0,1\right)$;
- parameter α; In order to select the value α that characterizes the spreading of the additional waypoints, two aspects must be considered: (a) a higher value for α will provide a higher unpredictability to the robot path; and (b) a higher value for α will need a higher robot speed to accomplish the mission in the required time (*t_max_*). Knowing the robot’s speed $v_{robot}$ (has to be as high as possible to increase the effectiveness of the method), we can compute an estimate of the maximum value for α (denoted with $\alpha_{max}$) by simulating a sufficiently long altered path (our method provides a random-looking sequence of waypoints, which basically depends on initialization values for ACM, so the covered distance may vary) to obtain a better approximation. Having the estimate $\alpha_{max}$ we can select a value using
  (8) $$\alpha <\alpha_{max}$$
  a practical example being given in Section 5.

The parameters set in this phase can be viewed as components of a secret-key that enables the ally entities (other robots from the same team or team’s coordinators) to have an accurate prediction of the robot’s location and its future movements.

**Phase 2: Offline preliminary calculations**

Before starting the patrol mission, we can derive two of the required parameters to initialize the robot path planning algorithm, namely the magnitude of speed *v*; and the number *M_i_* of additional waypoints that must be generated for each segment *P_i_P_i_*_+1_ of the trajectory:

- the magnitude of the speed *v* (average speed to cover the unaltered path) can be computed using:
  (9) $$v=\frac{d}{t_{max}}=\frac{\sum_{i=1}^{N-1}\sqrt{{\left(x_{i+1}-x_{i}\right)}^{2}+{\left(y_{i+1}-y_{i}\right)}^{2}}}{t_{max}}$$
  where *d* is the distance covered by the robot on the reference (unaltered) path and can be calculated by summing the length of all segments *P_i_P_i_*_+1_ with *i =* 1, …, *N* − 1;
- The number *M_i_* of additional waypoints is influenced by the length of the segment *P_i_P_i_*_+1_ and the magnitude of the speed v used to traverse this segment.
  (10) $$M_{i}=\lfloor \frac{\sqrt{{\left(x_{i+1}-x_{i}\right)}^{2}+{\left(y_{i+1}-y_{i}\right)}^{2}}}{v\cdot \tau }-1\rfloor =\lfloor \frac{\sqrt{{\left(x_{i+1}-x_{i}\right)}^{2}+{\left(y_{i+1}-y_{i}\right)}^{2}}}{v}-1\rfloor$$
  where the time τ to cover each of the $M_{i}$ subdivision of the segment *P_i_P_i_*_+1_ is equal to one time sample (τ=1), while the notation ⌊⌋ denotes the floor function, also known as greatest integer function.

**Phase 3: Mission accomplishment and online calculations**

In this stage, the robot is autonomously moving from one waypoint to another to accomplish its given missions. The additional waypoints, generated using the AWG algorithm, are inserted between original waypoints to obtain a deceptive path that emulates the protean behavior of prey animals when chased by predators. This stage normally includes the additional waypoints calculations, but if the original trajectory is short enough, these calculations can be done offline in the second phase.

The bioinspired robot motion can be implemented using the following pseudocode:

1. initialize {*P_i_, i =* 1, *…*, *N* }, ${\hat{x}}_{0},{\hat{y}}_{0}$, α, *t_max_* 2. compute v;   // Equation (9) is used 3. **for** (*i* = 1; *i* < *N*; *i*++) 4.  compute *M_i_*; //compute the number of additional waypoints for *P_i_P_i_*_+1_ segment using Equation (10) 5. start moving; 6. **for** (*i* = 1; *i* < *N*; *i*++){ 7. { 8.  [*Q_i_*_,1_,…*Q_i,M_*, ${\hat{x}}_{M}{}_{i}$, ${\hat{y}}_{M_{i}}$] = AWG(*P_i_*, *P_i_*_+1_, *M_i_*, ${\hat{x}}_{0}$, ${\hat{y}}_{0},$
*v*, α,); // generates additional the waypoints 9.  **for** (*k* = 1; *k* <= *M_i_*; *k*++) 10.   Move_towards_waypoint(*Q_i,k_*); //the robot is heading towards the additional waypoint 11.  Move_towards_waypoint(*P_i+_*_1_);  //the robot is heading towards the end of the segment 12.  ${\hat{x}}_{0}$ = ${\hat{x}}_{M}{}_{i}$; //prepare the initialization parameters for the following line segment 13.  ${\hat{y}}_{0}$ = ${\hat{y}}_{M}{}_{i}$; 14. }

In this pseudocode, the Move towards waypoint (P) function represents a motion planning and obstacle avoidance algorithm used to reach the destination P. In this regard, two types of approaches may be considered: global path planning and local path planning. Global path planning algorithms, such as Breadth-first search, Depth-first search, A*, Dijkstra’s algorithm, or Potential Field [37], consider completely known maps of the environment. On the other hand, the local path planning schemes that include bug-like algorithms [36,44,45], visibility binary tree algorithm [46] or methods based on perception-action map learning [47], use current local environment information and are designed to tackle the scenarios where the map of environment is unknown or dynamic. For exemplifying reasons, we will use an effective and generally applicable bug-like obstacle avoidance algorithm for unknown environments, named TangentBug [48]. Similar to other algorithms from the bug family [36,49], TangentBug provides paths made up of two motion types: motion-to-goal and boundary-following motion. In the case that no obstacle is encountered, the robot follows a straight line until it reaches the goal (motion-to-goal). In the case that an obstacle is encountered, the robot circumnavigates the obstacle until it finds a direct straight line to the goal (boundary-following).

In addition, the Move towards waypoint (P) function includes a procedure to drop the additional waypoints that fall inside obstacles either by directly verifying their position in case the robot knows the exact position, shape and size of the obstacles or by setting a time limit to reach them (e.g., two times higher than the time to reach it in an environment without obstacles) in case of unknown obstacles.

## 5. Simulation Results

For evaluating the effectiveness and applicability of our method, two extensive Matlab (version R2016b, MathWorks Inc., Natick, MA, USA) simulation case studies have been carried out. The first one exemplifies the proposed method in all its details, while the second one compares the method with the one based on fictive temporary obstacles generation provided by [35].

### 5.1. First Case Study

We considered a square workspace (18 m × 18 m), where we defined a basic octagonal patrolling contour described by the following nine waypoints placed in the vertices: *P*_1_(5, 0), *P*_2_(0, 5), *P*_3_(0, 10), *P*_4_(5, 15), *P*_5_(10, 15), P_6_(15, 10)*, P*_7_(15, 5), *P*_8_(10, 0) and *P*_9_(5, 0). The mobile robot is equipped with an obstacle detection sensor having a range of 0.5 m and is moving with a maximum velocity of 1.0 m/s. The robot is tasked to cover this closed contour in no more than *t_max_ =* 120 s. 

**Figure 6:**
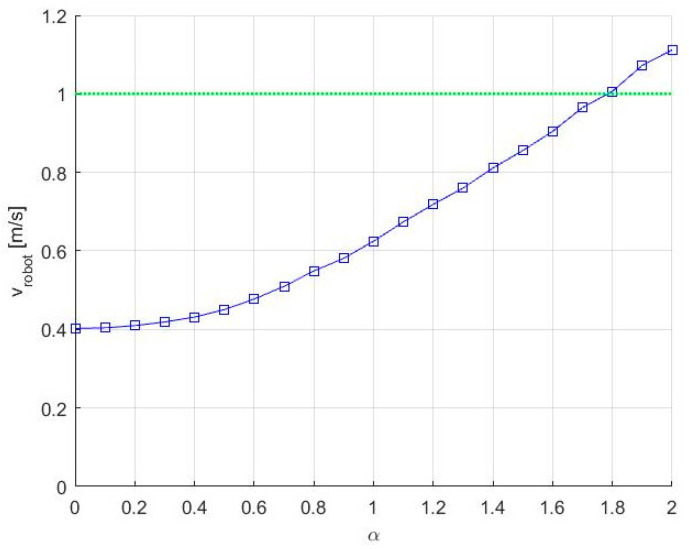
Selecting a suitable value for α.

**Figure 7:**
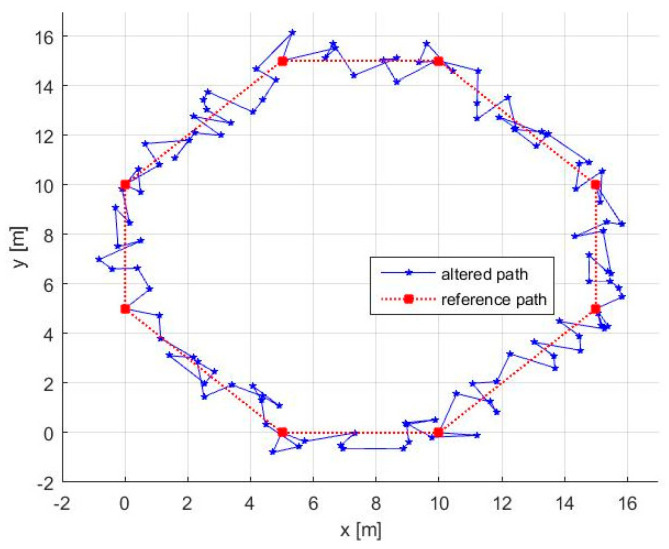
Altered path and reference path.

Knowing the initial set of waypoints *P_i_* and the time *t_max_*, in the initialization phase, we need to set the initial values for ACM and to select a suitable value for α. The initial values for ACM are set as $\left({\hat{x}}_{0},{\hat{y}}_{0}\right)=\left(0.4,\;0.644\right)$. For obtaining a suitable value α, in Figure 6, we represented the speed needed to cover the trajectories for different values of α. Because, in our simulation scenario, the robot’s maximum speed is 1.0 m/s we can choose α=1.7. It is worth mentioning that, for increasing the effectiveness of the method, α and also $v_{robot}$ must take values as high as possible while respecting the limitations imposed by terrain and robot’s remaining energy. Using these initialization parameters, we can follow the pseudocode to obtain the altered path presented in Figure 7, where the needed velocity for the robot is 0.9651.

**Figure 8:**
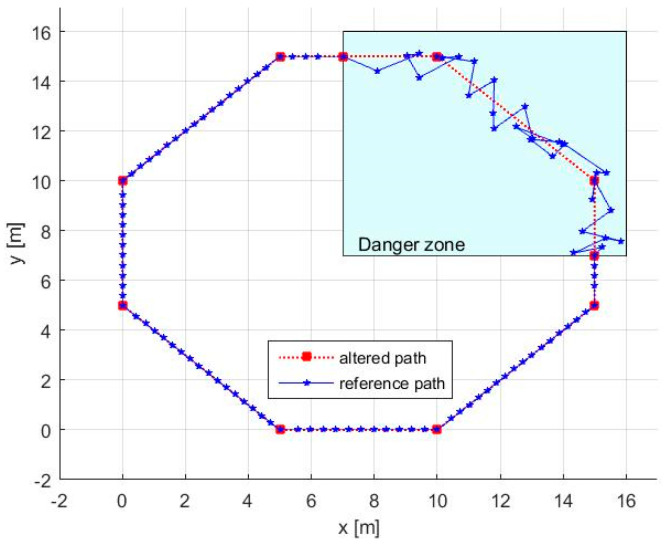
The path in danger and safety zones.

Remarks:

(a) As presented in Figure 7, in many circumstances, the altered path has a “go-backward” component. This can be observed anytime v<α, this inequality being a direct result of the way AWG was designed. In our case, using Equation (9), we obtain *v =* 0.4024, so the mentioned inequality is satisfied.
(b) Theoretically, the path always touches the initial waypoints. In practice, the robot has an allowed position error range, which means that the waypoints are considered as reached when the robot is within a circle around the waypoint with a radius smaller than the allowed position error.
(c) The method can simply deal with missions where only some parts of the path are under threat. In this case, we need: (i) to set initial waypoints anytime the robot enters or leaves the danger zone; and (ii) to generate additional waypoints using our method only for the segments that lay inside the danger zone (Figure 8).

**Figure 9:**
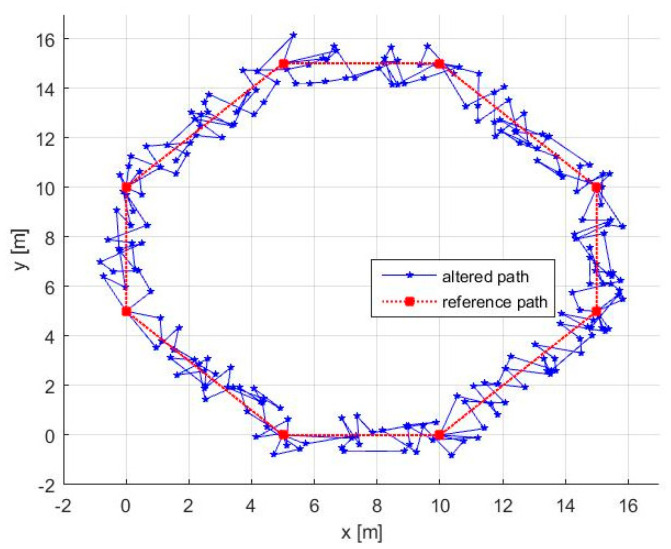
Three laps of the path.

To demonstrate the unpredictability of the path, in Figure 9, three consecutive laps are presented for the same octagonal contour, the use of ACM guaranteeing that the path cannot be periodic.

In order to numerically illustrate the unpredictability of the path, we used an ensemble of four metrics, which offers a holistic view about the effectiveness of the proposed method: mean absolute error (MAE), average number of direction changes (AMDC) in a given time interval, the mean absolute angle (MAA) for direction changes and the range of angles (RA) for direction changes.

An appropriate metric that can be used to evaluate the overall unpredictability of the robot path is the Mean Absolute Error (MAE) between two sequences of points: (a) the *L* equally distanced points $P_{l}^{*}\;\mathrm{with}\;l=1,\ldots ,\;L$ placed on the reference path $C^{*}$; and (b) the corresponding *L* time-equidistant points $P_{l}^{\prime }$ placed on the altered path $C^{\prime }$. This metric reflects the robot trajectory deviation from its reference path and is computed by the following formula:

(11) $$MAE\left(C^{*},C^{\prime }\right)=\frac{1}{L}\overset{L}{\underset{l=1}{\sum }}d\left(P_{l}^{*},P_{l}^{\prime }\right)$$

where $d\left(P_{l}^{*},P_{l}^{\prime }\right)$ is the Euclidean distance between the specified point $P_{l}^{*}$ of the altered path and the corresponding point $P_{l}^{\prime }$ on the reference contour (unaltered path). We say that the two points $P_{l}^{*},P_{l}^{\prime }$ are correspondent if the length of the reference path and of the altered path are divided by the two points in exactly the same ratio, respectively. The MAE metric described by (11) is particularly compelling for practical use because it facilitates standardized comparisons of candidate trajectories with the reference path. Its value is higher when the unpredictability of the altered path is higher.

**Figure 10:**
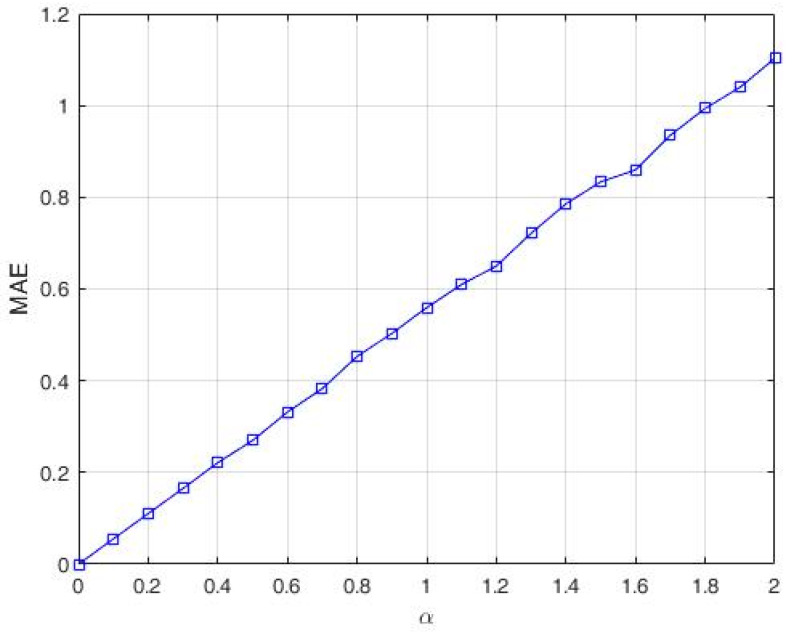
MAE metric for different values of α.

As expected, MAE, computed in *L* = 1440 points (12 laps, with 120 points per lap), increases linearly with α, as depicted in Figure 10.

In our case, where α=1.7, the value for the MAE metric obtained through simulations is 0.9352.

While MAE characterizes the long-term unpredictability of the path, the other three metrics that we employed (ANDC, MAA and RA) can provide valuable insights about protean behavior feature of the path. For our method, the average number of direction changes (ANDC) in a given time *t* is a direct result of selecting the *t_max_* and α parameters, while the range of angles (RA) for direction changes derives directly from the design of the method. In the specific conditions taken into account in the first case study, *ANDC =* 0.4024 direction changes per second and *RA =* (−*π*, *π*] radians.

Another metric that we used to evaluate the short-term unpredictability (i.e., the main characteristic of protean behavior) is the mean absolute angle (MAA) for direction changes and can be computed using the following equation:

(12) $$MAA=\frac{1}{K-1}\sum_{k=1}^{K-1}\left|\vartheta_{k+1}-\vartheta_{k}\right|,$$

where *K* is the number of the segments in the altered path, while $\vartheta_{k}$ is the angle between a segment and the *x*-axis. This metric reflects the uncertainty in predicting the next direction on which the robot will evolve. For the trajectory depicted in Figure 7, we obtained *MAA =* 0.7815 radians.

### 5.2. Second Case Study

This case study is aimed to analyze the proposed AWG-based method in the presence of fixed obstacles and also to compare this method with the one based on fictive temporary obstacles (FTOs) generation [35]. For this, we considered a square workspace (12 × 12 square meters), where we marked a reference contour described by four initial waypoints having the following coordinates: *P*_1_(1, 1), *P*_2_(1, 11), *P*_3_(11, 11) and *P*_4_(11, 1). The mobile robot is moving with a constant speed of 1 m/s and uses a bug-like obstacle avoiding mechanism (i.e., TangentBug) based on an obstacle detection sensor having a range of 0.5 m.

**Figure 11:**
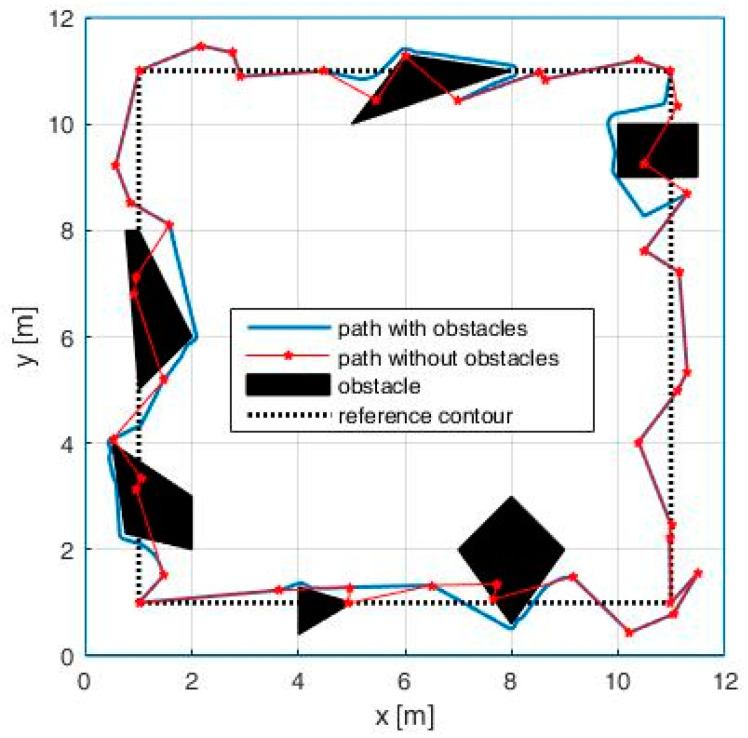
Impact of the obstacles.

In order to study the proposed path-planning method in a more complex environment, we placed a set of fixed obstacles having various shapes and sizes inside the workspace (Figure 11). In this context, as discussed at the end of Section 4, the robot must drop any additional waypoint that falls inside the obstacles. This procedure is very simple if the robot knows the exact position, shape and size of the obstacles. In the case that the obstacles are unknown, we can pursue the following procedure: an additional waypoint is left aside if it is not reached in a given time (e.g., two times higher than the time to reach it in an environment without obstacles).

From Figure 11, we can see that, in the proximity of small size obstacles, the unpredictability of the trajectory remains high, while, in the case of large obstacles, where more additional waypoints are dropped, the unpredictability gets lower, the robot being forced to take a circumnavigating path around obstacles.

The second objective of this case study is to compare the proposed AWG-based method with the one based on FTOs, which adopts a different strategy to impart protean behavior to the patrol robot. This strategy uses the intrinsic obstacle avoidance mechanism of the robot not only to circumvent real obstacles but also to avoid a random-looking series of fictive and temporary obstacles. In this case study, the two robot’s paths are generated as follows: (i) the path generated using FTOs considered the following parameters of the algorithm: a FTO appearance time between 2 and 20 s, a FTO active time between 0.3 and 2.5 s and the initialization values $x_{0}=0.18$ and ; and (ii) the AWG-based robot path uses α=1.3 to assure the same number of direction changes per lap (ANDC = 0.4024).

**Figure 12:**
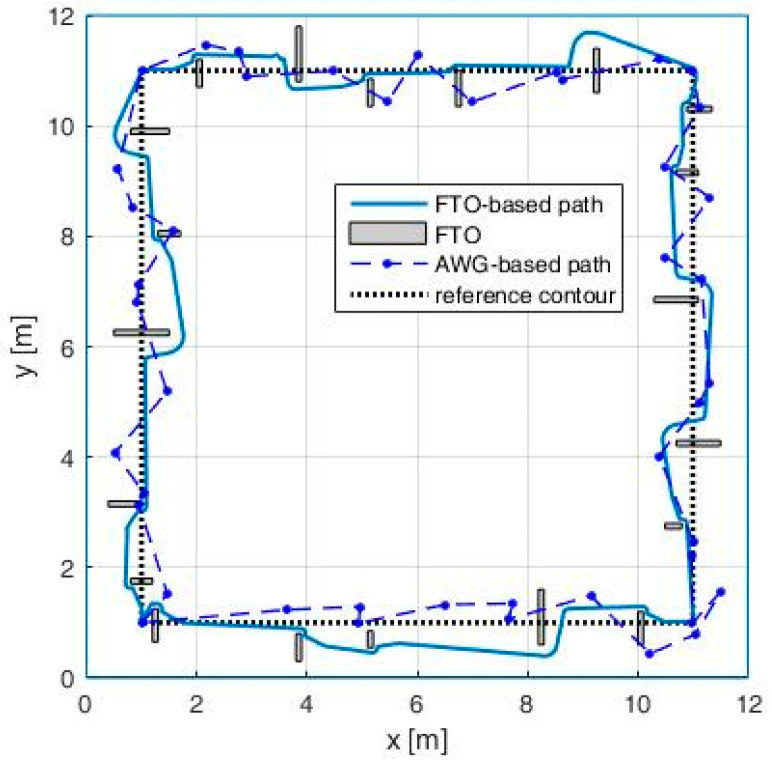
Impact of FTOs above robot’s path.

In the first scenario, we considered an obstacle-free workspace and we represented the two misleading paths generated by both methods (Figure 12). We computed the mentioned metrics for the two paths obtaining the following results: for the AWG-based path, we have *MAE =* 0.7120, *RA =* (−*π*, *π*] and *MAA =* 0.7854, while, for the FTO-based path, we have *MAE =* 1.5769, *RA* = [−*π*/2, *π*/2] and *MAA* = 0.3681. These results show two metrics favorable for the AWG-based method and one metric (MAE) favorable for the FTO-based method, while the fourth metric is the same (*ANDC =* 0.4024) for both methods. Overall, based on the values previously presented for the four considered metrics and based on the visual analysis of the simulated trajectories, the AWG based method generates a path, which better emulates the zig-zag movements that characterizes the protean behavior due to two reasons: (i) the path has sudden and more abrupt direction changes; and (ii) path segments with go-backward components are also possible.

**Figure 13:**
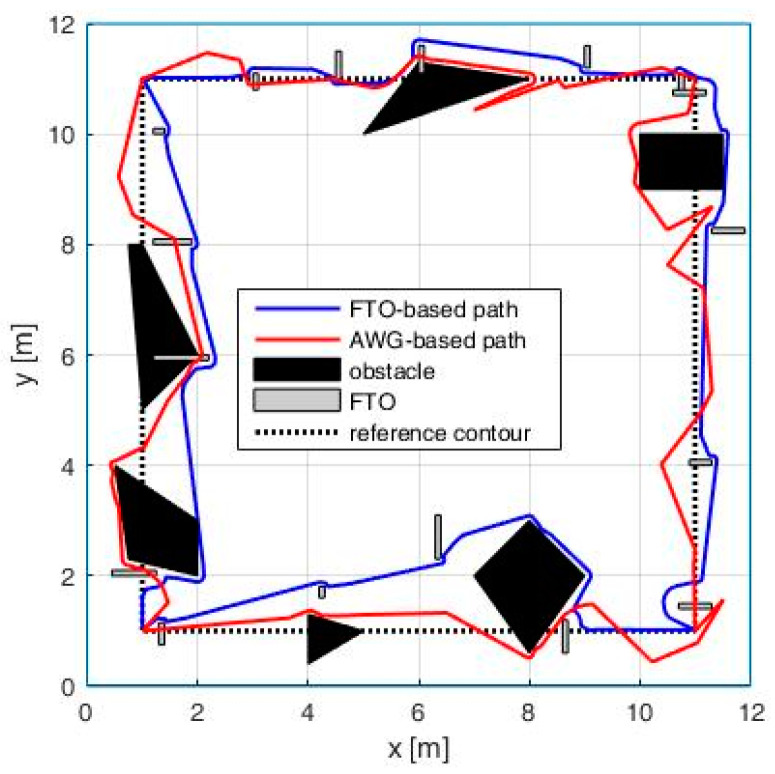
Impact of the obstacles on the path.

In the second scenario, the two methods are compared in a workspace containing different fixed obstacles (Figure 13). While for small size obstacles the AWG-based method is still the best variant, for large obstacles, the FTO-based method offers greater path unpredictability (the FTO are applied even when the robot circumnavigates an obstacle), so a mix between the two methods can be a feasible solution for these situations.

## 6. Conclusions

The paper provides a new bio-inspired method to generate deceptive paths for mobile robots accomplishing patrolling missions under adversarial threat. Using the 2D Arnold cat map as a primary source of positional entropy, the reference path is altered without compromising the overall goals of the patrolling mission. The obtained misleading trajectory emulates an unpredictable zig-zag motion that is similar to the one developed by individual pray animals when chased by predators. Compared with the method based on fictive-temporary obstacle generation, the present method offers superior results in environments without or with small/medium size obstacles. Furthermore, the paper suggests the idea of mixing the two methods to cope with complex environments where large obstacles can also be encountered.
